# Diminished neutralization responses towards SARS-CoV-2 Omicron VoC after mRNA or vector-based COVID-19 vaccinations

**DOI:** 10.1038/s41598-022-22552-y

**Published:** 2022-11-18

**Authors:** Henning Jacobsen, Monika Strengert, Henrike Maaß, Mario Alberto Ynga Durand, Maeva Katzmarzyk, Barbora Kessel, Manuela Harries, Ulfert Rand, Leila Abassi, Yeonsu Kim, Tatjana Lüddecke, Kristin Metzdorf, Pilar Hernandez, Julia Ortmann, Jana-Kristin Heise, Stefanie Castell, Daniela Gornyk, Stephan Glöckner, Vanessa Melhorn, Yvonne Kemmling, Berit Lange, Alex Dulovic, Patrick Marsall, Julia Häring, Daniel Junker, Nicole Schneiderhan-Marra, Markus Hoffmann, Stefan Pöhlmann, Gérard Krause, Luka Cicin-Sain

**Affiliations:** 1 Department of Viral Immunology, Helmholtz Centre for Infection Research, Braunschweig, Germany; 2 Department of Epidemiology, Helmholtz Centre for Infection Research, Braunschweig, Germany; 3 TWINCORE, Centre for Experimental and Clinical Infection Research, Joint Venture of the Hannover Medical School and the Helmholtz Centre for Infection Research, Hannover, Germany; 4 NMI Natural and Medical Sciences Institute at the University of Tübingen, Reutlingen, Germany; 5 German Centre for Infection Research (DZIF), Partner Site Hannover-Braunschweig, Braunschweig, Germany; 6 Deutsches Primatenzentrum, Leibniz-Institut Für Primatenforschung, Göttingen, Germany; 7 Faculty of Biology and Psychology, Georg-August-University, Göttingen, Germany; 8 Centre for Individualized Infection Medicine (CIIM), Joint Venture of Helmholtz Centre for Infection Research and Medical School Hannover, Inhoffenstraße 7, 38124 Braunschweig, Germany

**Keywords:** Immune evasion, Infection, Viral infection, Vaccines, RNA vaccines, Immunology, SARS-CoV-2, Microbiology, Infectious-disease diagnostics, Pathogens, Vaccines, Virology

## Abstract

SARS-CoV-2 variants accumulating immune escape mutations provide a significant risk to vaccine-induced protection against infection. The novel variant of concern (VoC) Omicron BA.1 and its sub-lineages have the largest number of amino acid alterations in its Spike protein to date. Thus, they may efficiently escape recognition by neutralizing antibodies, allowing breakthrough infections in convalescent and vaccinated individuals in particular in those who have only received a primary immunization scheme. We analyzed neutralization activity of sera from individuals after vaccination with all mRNA-, vector- or heterologous immunization schemes currently available in Europe by in vitro neutralization assay at peak response towards SARS-CoV-2 B.1, Omicron sub-lineages BA.1, BA.2, BA.2.12.1, BA.3, BA.4/5, Beta and Delta pseudotypes and also provide longitudinal follow-up data from BNT162b2 vaccinees. All vaccines apart from Ad26.CoV2.S showed high levels of responder rates (96–100%) towards the SARS-CoV-2 B.1 isolate, and minor to moderate reductions in neutralizing Beta and Delta VoC pseudotypes. The novel Omicron variant and its sub-lineages had the biggest impact, both in terms of response rates and neutralization titers. Only mRNA-1273 showed a 100% response rate to Omicron BA.1 and induced the highest level of neutralizing antibody titers, followed by heterologous prime-boost approaches. Homologous BNT162b2 vaccination, vector-based AZD1222 and Ad26.CoV2.S performed less well with peak responder rates of 48%, 56% and 9%, respectively. However, Omicron responder rates in BNT162b2 recipients were maintained in our six month longitudinal follow-up indicating that individuals with cross-protection against Omicron maintain it over time. Overall, our data strongly argue for booster doses in individuals who were previously vaccinated with BNT162b2, or a vector-based primary immunization scheme.

## Introduction

Since its emergence in late 2019, SARS-CoV-2 has caused a pandemic with several hundred million confirmed infections and dramatic mortality^1^. While multiple vaccines have been developed with unprecedented speed and were successfully deployed to limit the burden of COVID-19, it became quickly apparent that novel SARS-CoV-2 variants had evolved, mainly in areas of high virus prevalence. Some of those emerging variants (Variants of Concern, VoC) have accumulated mutations in several proteins, including the immuno-dominant surface-exposed Spike protein, which increase virus transmissibility and/or promote evasion from the host immune response^2–4^. To date, neutralizing antibodies have been found to be a promising correlate of protection against infection for previous SARS-CoV-2 variants, but a precise correlate of protection on both cellular and humoral level is still lacking^5,6^. Until the end of 2021, immune escape was most pronounced in SARS-CoV-2 variants Beta (B.1.351) and to a lesser extent in Delta (B.1.617.2). Importantly, vaccine protection against the Beta and Delta variants seems to be reduced only against infection, but not severe COVID-19 disease or death^7^. However, the emergence of the B.1.1.529 variant (Omicron) in South Africa in late 2021 has raised strong concerns as its unusually high number of amino acid alterations in the Spike protein likely contributes to an increased reinfection risk or breakthrough infections following vaccination^8^. By now, a series of studies have addressed the impact of Omicron on vaccination- or infection-induced antibody neutralization by using either live-, pseudo-virus neutralization or in vitro binding assays with samples from convalescent and vaccinated individuals^9–17^. These studies have shown clear losses of neutralization capacity against the BA.1 Omicron variant but did not comprehensively address antibody responses in various vaccination regimens or over time and provide only information on post-boost cohorts but not for cohorts after primary immunization. Additionally, several sub-variants of Omicron have emerged and replaced the original BA.1 variant across the world. As of now, only few studies provide comprehensive data sets against these sub-variants, especially in primary vaccination settings. We provide here such a comprehensive assessment of vaccination schemes approved in the European Union and the UK, using an Omicron (BA.1), Beta, Delta or Wuhan B.1 pseudo-neutralization assay at peak response after approximately four weeks and in a longitudinal six month follow-up for BNT162b2. Additionally, we provide pseudo-virus neutralization data against other clinically relevant sub-variants of Omicron (BA.2, BA.2.12.1, BA.3, BA.4 and BA.5) in a subset of participants from all vaccine groups.

## Methods

### Sample collection and ethics statement

Serum samples analyzed in this study originate from vaccinated participants of the multi-local and serial cross-sectional prevalence study on antibodies against SARS-CoV-2 in Germany (MuSPAD), a population-based SARS-CoV-2 seroprevalence study conducted in eight regions of Germany from July 2020 to August 2021. The study was approved by the Ethics Committee of the Hannover Medical School (9086_BO_S_2020) and was in line with the Declaration of Helsinki. Briefly, MuSPAD is a successive cross-sectional study where certain locations were sampled longitudinally within a 3–4 month interval^18^. Recruitment of eligible participants (> 18 years) was based on age- and sex-stratified random sampling with information provided by the respective local residents’ registration offices. Basic sociodemographic data and information on pre-existing medical conditions including a previous SARS-CoV-2 infection or vaccination are self-reported and were documented with the eResearch system PIA (Prospective Monitoring and Management-App) at the study center. Peripheral blood was obtained by venipuncture using a serum gel S-Monovette (Sarstedt) and further processed according to the manufacturer`s instructions. Serum was then aliquoted at the German Red Cross Institute of Transfusion Medicine and Immunohematology and transported on dry ice to the Hannover Unified Biobank for long-term storage.

For this study, we selected samples from 144 vaccinees from the available MuSPAD sample pool to contain mRNA, vector and heterologous (commonly referred to as “mix and match”) immunization schemes. Selection was primarily based on a consistent 21–35 day ΔT range from a complete vaccination until sampling with comparable age and sex distribution between vaccination schemes, if available paired longitudinal follow-up samples were selected of samples taken at peak response. None of the selected donors reported a positive SARS-CoV-2 antigen or PCR test result when questioned at the study center at the time of blood draw. Additionally, all collected serum samples were non-reactive for nucleocapsid-specific IgG as determined with MULTICOV-AB, a previously published multiplex immunoassay that contains SARS-CoV-2 Spike and nucleocapsid antigens (Supplementary Table S8,^19^), therefore excluding confounders due to infections superposed on vaccination in our cohort. Vaccination details with basic sociodemographic information and pre-existing conditions such as hypertension, cardiovascular disease, diabetes, lung disease, immunosuppression or cancer of participants are provided in Table 1 and Supplementary Table S1. As controls, the first WHO International Standard for human anti-SARS-CoV-2 immunoglobulin (code: 20/136) from the National Institute for Biological Standards and Control (NIBSC) or pre-pandemic sera samples from an anonymized Hepatitis A and Influenza virus vaccination response study at the Helmholtz Centre for Infection Research in 2014 (Hannover Medical School Ethics Committee approval number 2198–2021) were used (Supplementary Table S8).

**Table 1:** Sample characteristics.

Sample cohort (n)	ΔT (days) post-complete vaccination (mean, SD)	ΔT (days) between doses (mean, SD)	Age (years), median (IQR)	Sex (n, %)	
				Female	Male
one-dose Ad26.CoV2.S^ (23)^	53.8 (17.1)	na	61 (53–68)	14 (60.9)	9 (39.1)
two-dose AZD1222^ (25)^	28.7 (2.7)	76.7 (3.1)	64 (62–66)	14 (56.0)	11 (44.0)
first dose AZD1222, second dose BNT162b2^ (25)^	25.3 (6.1)	70.8 (16.8)	67 (57–70)	11 (44.0)	14 (56.0)
first dose AZD1222, second dose mRNA-1273^ (24)^	23.2 (12.8)	69.6 (16.9)	68 (65–71)	15 (62.5)	9 (37.5)
two-dose mRNA-1273^ (24)^	31.7 (5.6)	30.7 (5.4)	56 (54–69)	15 (62.5)	9 (37.5)
two-dose BNT162b2 T1 *^ (23)^	29.5 (7.3)	21.2 (1.0)	51 (41–58)	18 (78.3)	5 (21.7)
two-dose BNT162b2 T2 *^ (23)^	174.0 (14.2)				

na. not applicable.

*Two-dose BNT162b2 T1 and two-dose BNT162b2 T2 are paired longitudinal samples.

### Cell culture

Vero E6 (ATCC CRL-1586), and 293 T (DSMZ ACC-635) were maintained in DMEM medium supplemented with 10% fetal bovine serum (FBS), 2 mM L-glutamine, 100 U/ml penicillin and 100 µg/ml streptomycin at 37 °C in a 5% CO_2_ atmosphere. All cell lines used within this study were below a passage of 50 and were regularly checked for mycoplasma contamination. Transfection of 293 T cells was performed using calcium-phosphate.

### Plasmids

Plasmids encoding SARS-CoV-2 Spike B.1 (human codon optimized, 18 amino acid truncation at C-terminus) and SARS-CoV-2 spike of Beta (B.1.351) and Delta (B.1.617.2) have been previously reported^20–22^. The expression vectors for SARS-CoV-2 Spike of Omicron BA.1 (based on isolate hCoV-19/Botswana/R40B58_BHP_3321001245/2021; GISAID Accession ID: EPI_ISL_6640919), BA.2 (based on isolate hCoV-19/England/PHEC-4G0AFZF7/2021; GISAID Accession ID: EPI_ISL_8738174), BA.2.12.1 (based on isolate hCoV-19/USA/FL-CDC-ASC210848866/2022; GISAID Accession ID: EPI_ISL_12028907), BA.3 (based on isolate hCoV-19/South Africa/NICD-N25677/2021; GISAID Accession ID: EPI_ISL_8801154) and BA.4/5 (based on isolate hCoV-19/England/LSPA-3C01A75/2022 [BA.4] and hCoV-19/France/CVL-IPP25260/2022 [BA.5]; GISAID Accession ID: EPI_ISL_11550739 [BA.4] and EPI_ISL_12029894 [BA.5])were generated by Gibson assembly^23^. All plasmids were sequence-confirmed by Sanger sequencing prior to use. Supplementary Table S2 provides an overview of Spike protein amino acid mutations used for SARS-CoV-2 pseudotype construction compared to the parental strain B.1.

### Pseudotyping

Generation of rhabdoviral pseudotypes harboring SARS-CoV-2 Spike proteins was performed as described^24^. In brief, 293 T cells were transfected with pCG1 plasmids expressing different SARS-CoV-2 Spike proteins, using calcium-phosphate. 24 h post transfection, cells were infected with a replication-deficient reporter VSV-G (VSV ∗ ΔG-Fluc) at an multiplicity of infection (MOI) of 3 for 1 h at 37 °C^25^. Cells were washed once with PBS and medium containing anti-VSV-G antibody (culture supernatant from L1-hybridoma cells) was added to neutralize residual input virus. The cell culture supernatant was harvested after 16 h, and cellular debris was removed by centrifugation at 2000 g for 5 min at 4 °C. Aliquots were stored at − 80 °C until use.

### Neutralization assay

For pseudo-virus neutralization, serum samples and controls were heat-inactivated at 56 °C for 30 min. Thawed samples and controls were stored at 4 °C for no longer than 48 h, prior to use. In a 96-well microtiter plate, serum samples were two-fold serially diluted in cell culture medium (DMEM, 5% FBS, 1% P/S, 1% L-Glu) with a dilution range of 1:10 to 1:5120. Pre-diluted samples were incubated with an equal volume of Spike protein-bearing viral particles (approximately 200–500 (fluorescence forming units (ffu)/well) at 37 °C for 1 h. After incubation, the sample-virus mixture was transferred to VeroE6 cells at 100% confluence which were seeded the day before. Cells were incubated at 37 °C for 24 ± 2 h, while infected cells were visualized using an IncuCyte S3 (Sartorius) performing whole-well scans (4x) in phase contrast and green fluorescence settings. Automated segmentation and counting of fluorescent foci defined as green fluorescent protein (GFP)^+^-single cells was performed using the IncuCyte GUI software (versions 2019B Rev1 and 2021B). Raw data were plotted in GraphPad prism version 9.0.2 and FRNT (focus reduction neutralization titer)_50_ was calculated with a variable slope, four-parameter regression analysis. Non-responders were defined as subjects with undetectable neutralization titers at an initial serum dilution of 1:10. FRNT_50_ values of those individuals were arbitrarily set to 1. All experiments were performed with internal standard controls (pool of all tested sera), negative controls and virus-only controls to assess the nominal virus input for every single measurement.

### Data analysis and statistics

Initial results collation and matching to metadata was done in Excel 2016 and R 4.1.0. Statistical calculations and graphs were generated using GraphPad Prism version 9.0.2 for Windows (GraphPad Software). Figure panels were generated using Inkscape 1.2.1. For analysis of neutralization assay results, a Shapiro–Wilk test was used to determine normality. Focus Reduction Neutralization titer with a 50% neutralization cut-off (FRNT_50_) was calculated using a four-parameter regression analysis function. FRNT_50_ values from non-responders were set to 1 for graphical presentation only. A non-parametric Friedman's test followed by Dunn's multiple comparison analysis was used to compare neutralization results to different viruses in a pair-wise manner for matched samples. Two-tailed Wilcoxon matched-pairs signed rank test was used to compare neutralization of longitudinal results. Spearman’s ρ was used for correlation analysis of FRNT_50_ values and antibody titers previously measured as part of^26^. A p-value of less than 0.05 was considered statistically significant.

### Role of the funders

This work was financially supported by the Initiative and Networking Fund of the Helmholtz Association of German Research Centres through projects “Virological and immunological determinants of COVID-19 pathogenesis–lessons to get prepared for future pandemics (KA1-Co-02 “COVIPA”) to LCS and “The Helmholtz Epidemiologic Response against the COVID-19 Pandemic” (SO-96 “HZEpiAdHoc”) to GK, by the Deutsche Forschungsgemeinschaft (DFG, German Research Foundation) under Germany’s Excellence Strategy EXC 2155 “RESIST” Project ID 39087428 to LCS, and intramural funds of the Helmholtz Centre for Infection Research. SP was supported by BMBF (01KI2006D, 01KI20328A, 01KX2021), the Ministry for Science and Culture of Lower Saxony (14-76,103-184, MWK HZI COVID-19) and the German Research Foundation (DFG; PO 716/11-1, PO 716/14-1). The funders had no role in study design, data collection, data analysis, interpretation, writing or submission of the manuscript. All authors had complete access to the data and hold responsibility for the decision to submit for publication.

### Ethics statement

The study was approved by the Ethics Committee of the Hannover Medical School (9086_BO_S_2020). All participants provided written informed consent prior to study start.

## Results

**Figure 1:**
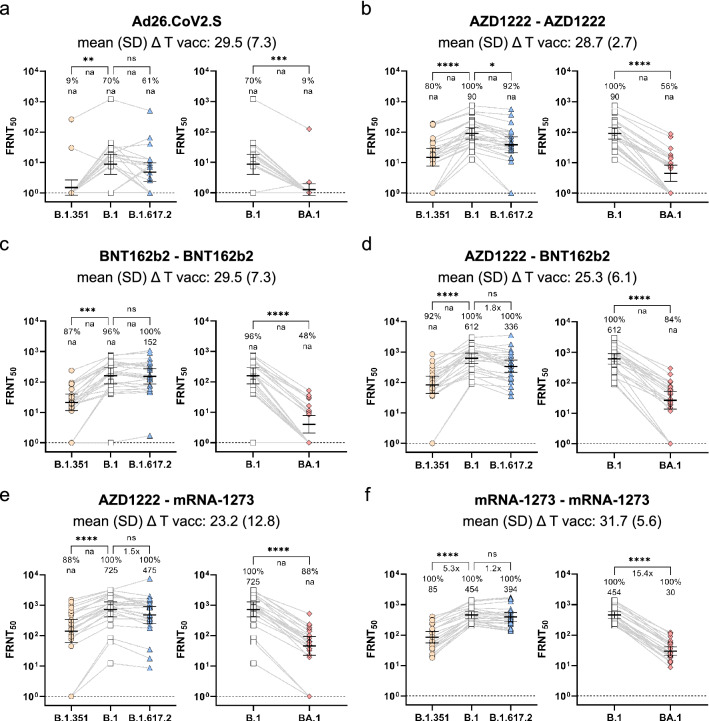
Impact of SARS-CoV-2 vaccination schemes on neutralization response towards the Omicron BA.1 variant. Vaccination-induced neutralization potency against Omicron (BA.1), Beta (B.1.351), Delta (B.1.617.2) or Wuhan (B.1) pseudotypes was measured in individuals who received a vector-based vaccination with single dose Ad26.CoV2.S (n = 23, **a**), two doses of AZD1222 (n = 25, **b**), two doses of mRNA vaccine BNT162b2 (n = 23, **c**), a heterologous two-dose vaccination with AZD1222-BNT162b2 (n = 25, **d**) or AZD1222-mRNA-1273 (n = 24, **e**), or two doses of mRNA vaccine mRNA-1273 (n = 24, **f**) after the indicated time periods following the last dose. FRNT_50_ data is expressed for each serum sample, bold horizontal lines and whiskers are geometric means with 95% CI. Interconnecting lines represent sample data from the same donor. Non-neutralizing sample values were arbitrarily set to 1 for presentation purposes, indicated by a dashed line. Fold change in neutralization potency between SARS-CoV-2 B.1 and VoC pseudotypes is shown below *p*-values. Percentage (%) responder rates and FRNT_50_ geometric mean titers (GMT) per SARS-CoV-2 pseudotype are shown above the individual measurements. Fold-change in neutralization potency and GMTs for SARS-CoV-2 pseudotypes are only calculated for groups where all samples had a detectable neutralizing activity, or else not applicable (na) is stated. Time between sampling and full vaccination in days is displayed as mean and SD below the vaccination scheme. Statistical analysis was performed by paired non-parametric Friedman's test followed by a Dunn's multiple comparison analysis. Statistical significance was defined by a value of * < 0.05; ** < 0.01; *** < 0.001; **** < 0.0001.

Neutralization responses towards B.1, BA.1, B.1.351, and B.1.617.2 Spike-expressing rhabdoviral pseudotypes were analyzed in serum samples from 144 individuals vaccinated with either a single dose of Ad26.COV2.S, homologous two-dose BNT162b2, mRNA-1273 or AZD1222 vaccination, or heterologous AZD1222-BNT162b2 or AZD1222-mRNA-1273 vaccination at peak response, approximately four weeks after the last dose. While the WHO international standard serum showed detectable neutralization against all variants including Omicron BA.1, showing a good sensitivity of our assay, compared to previous studies^27^, pre-pandemic control sera from 2014 (n = 4) showed no measurable neutralization levels (Supplementary Table S8). Neutralization potency towards Beta VoC pseudotypes were clearly reduced for all vaccination schemes, however the strongest effect across all samples tested were found against Omicron BA.1 (Fig. 1). Vaccination with vector-based Ad26.CoV2.S performed least well (Fig. 1a), with only 70% responders against the B.1 variant, 9% for the Beta B.1.351 and 9% for Omicron BA.1. Homologous vaccination with either AZD1222 or BNT162b2 performed better against Omicron BA.1, with 56% or 48% responders, respectively (Fig. 1b, 1c). Heterologous immunization with these two vaccines (AZD1222-BNT162b2) showed a response rate of 84% (Fig. 1d). Heterologous vaccination with AZD1222-mRNA-1273 had a similar response rate of 88% (Fig. 1e), but homologous immunization with mRNA-1273 had the highest Omicron BA.1 response rate of 100% (Fig. 1f). Non-parametrical statistical comparisons showed a highly significant reduction in serum titers when Omicron BA.1 neutralization was compared to B.1 for all vaccination schemes (Fig. 1a–e). While we measured pseudotype-mediated antibody neutralization, antibody titers towards the parental SARS-CoV-2 Spike and RBD B.1 antigens and responder rates were determined previously using MULTICOV-AB, a multiplex-based SARS-CoV-2 immunoassay^26^. We found a good correlation between the responder rates towards the Wuhan B.1 strain across the two assay systems with only one-dose Ad26.CoV2.S vaccinees having more diverging results of 82.6% serological responders, but only a 70% response rate in the B.1 pseudo-virus neutralization assay (Supplementary Table S3 and S8). There was no strong tendency of age, sex, or pre-existing medical conditions to modify the responder status against Omicron BA.1 in our cohort (Supplementary Table S1 and S8) as already previously shown for other VoCs when analyzing humoral response profiles^26^. To assess the impact of immune escape with more detail, we focused on the responders and compared geometric means of their FRNT_50_ titers (GMT). Importantly, fold changes for groups that included non-responders are not provided in Fig. 1 because this approach can lead to highly artificial results and possibly over-interpretation as illustrated by the calculations summarized in Supplementary Table S4. We therefore present the percentage of responders as primary outcome and provide GMT fold changes where calculation is reasonable (100% responders in both arms). Furthermore, for each vaccination regimen, we defined the responder subgroups (excluding non-responders defined as subjects with undetectable neutralization titers at an initial 1:10 serum dilution in either group) and compared the fold-reduction for titers that could be quantified (see Table 2). In these subsets, we observed an approximate 10–15-fold reduction in GMT against Omicron BA.1 for most vaccinations, except BNT162b2, where the reduction was 22-fold at peak response. This was consistent with the high frequency of 48% non-responders in this subset, additionally arguing for weaker protection against infection with the Omicron BA.1 variant in this cohort.

**Table 2:** Geometric means of responses and fold reduction in paired samples of Omicron responder subsets.

Paired responders in sample cohort (n; [% of total n per vaccination scheme])	GMT (95% CI) of paired responders across indicated variants			
	B.1	Beta B.1.351	Delta B.1.617.2	Omicron BA.1
One-dose Ad26.CoV2.S * (1; [4.4])	na	na	na	na
Two-dose AZD1222 (14; [56.0])	155.80 (96.60–251.20)	40.98 (23.66–70.97)	83.52 (49.68–140.40)	14.39 (8.92–23.23)
First dose AZD1222, second dose BNT162b2 (21; [84.0])	791.90 (558.90–1122.00)	132.0 (87.94–198.00)	469.50 (316.00–697.50)	49.72 (34.00–72.71)
First dose AZD1222, second dose mRNA-1273 (21; [87.5])	1098.00 (793.80–1518.00)	284.20 (183.20–440.90)	760.70 (513.60–1127.00)	78.68 (54.36–113.90)
Two-dose mRNA-1273 (24; [100.0])	453.80 (351.2–586.4)	85.48 (55.45–131.80)	393.50 (284.4–544.4)	29.50 (21.58–40.33)
Two-dose BNT162b2 T1 (11; [47.8]) **	406.80 (300.90–550.00)	54.41 (32.19–91.99)	371.00 (243.30–565.50)	18.21 (11.83–28.03)
Two-dose BNT162b2 T2 (13; [56.2]) **	98.77 (68.41–142.60)	33.07 (21.25–51.45)	82.63 (53.60–127.4)	14.22 (9.74–20.75)

Paired responders in sample cohort (n; [% of total n per vaccination scheme])	Fold reduction in GMT of paired responders across indicated variants			
	B.1	Beta B.1.351	Delta B.1.617.2	Omicron BA.1
One-dose Ad26.CoV2.S * (1; [4.4])	–	na	na	na
Two-dose AZD1222 (14; [56.0])	–	3.80	1.87	10.83
First dose AZD1222, second dose BNT162b2 (21; [84.0])	–	6.00	1.69	15.93
First dose AZD1222, second dose mRNA-1273 (21; [87.5])	–	3.86	1.44	13.96
Two-dose mRNA-1273 (24; [100.0])	–	5.31	1.15	15.38
Two-dose BNT162b2 T1 (11; [47.8]) **	–	7.48	1.10	22.34
Two-dose BNT162b2 T2 (13; [56.2]) **	–	2.99	1.20	6.95

*Only one individual showed detectable neutralization titers in the Omicron BA.1 assay.

**Two-dose BNT162b2 T1 and two-dose BNT162b2 T2 are paired longitudinal samples.

**Figure 2:**
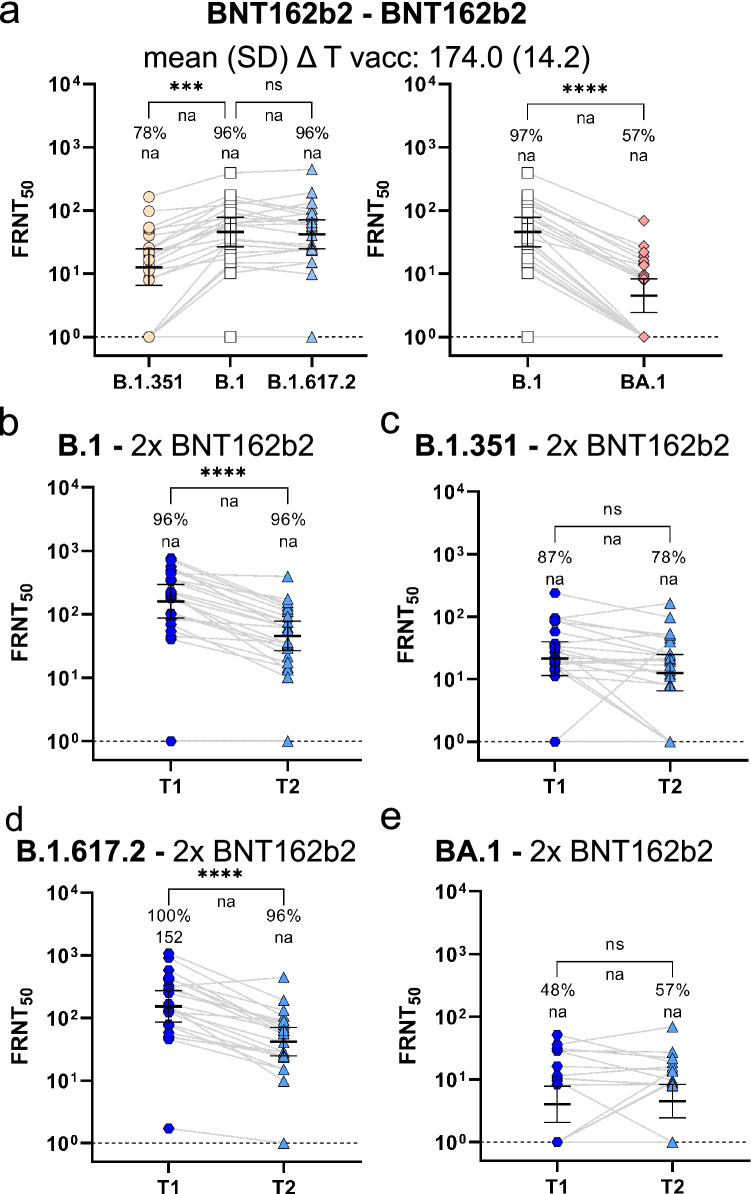
Longitudinal BNT162b2 neutralization response towards SARS-CoV-2 VoC Omicron sub-lineage BA.1. Neutralization capacity towards SARS-CoV-2 Omicron (BA.1), Beta (B.1.351), Delta (B.1.617.2) or Wuhan (B.1) pseudotypes was analyzed approximately six months after a two-dose BNT162b2 vaccination (n = 23), (**a**). Neutralization kinetic of paired longitudinal samples towards SARS-CoV-2 B.1 (**b**), Beta (**c**), Delta (**d**) and Omicron BA.1 (**e**) pseudotypes is shown between T1 (n = 23; mean (SD) ΔT after last dose: 29.5 (7.3)) and T2 (n = 23; mean (SD) ΔT after last dose: 174.0 (14.2)). Peak neutralization responses of BNT162b2-vaccinated individuals from Fig. 1c are displayed again for clarity and comparison (b-e). FRNT_50_ data is expressed for each serum sample, bold horizontal lines and whiskers are geometric means with 95% CI. Interconnecting lines represent sample data from the same donor (**a**–**e**). Non-neutralizing samples were arbitrarily set to 1 for presentation purposes, indicated by a dashed line. Fold-change in neutralization potency between SARS-CoV-2 B.1 and VoC pseudotypes is shown below *p*-values. Percentage (%) responder rates and FRNT_50_ geometric mean titers (GMT) per SARS-CoV-2 pseudotype are shown above the individual measurements. Fold change in neutralization potency and GMTs for SARS-CoV-2 pseudotypes are only calculated for groups where all samples had a detectable neutralizing activity, or else not applicable (na) is stated. Statistical analysis was performed by paired non-parametric Friedman's test followed by a Dunn's multiple comparison analysis (**a**) or a by two-tailed Wilcoxon matched-pairs signed rank test (**b**–**e**). Statistical significance was defined by a value of * < 0.05; ** < 0.01; *** < 0.001; **** < 0.0001.

We tested the neutralization potency in BNT162b2 recipients also at approximately six months post immunization as BNT162b2 continues to be the most commonly used vaccine in Germany making up 75% of all distributed doses towards the end of December 2021^28^ and in September 2022 with 74% of all applied doses^29^. Similar to the peak responses, we observed a significantly weaker neutralization of the Omicron BA.1 compared to the B.1 pseudotype and only 48% responders against Omicron BA.1 (Fig. 2a). Beta neutralization was slightly reduced, whereas Delta neutralization was at the same levels as B.1 at the late time point (Fig. 2a). To understand the longitudinal dynamic of humoral immunity, we used paired sera from BNT162b2 vaccine recipients at four weeks (already shown in Fig. 1c) and at approximately six months post second dose which allowed us to compare intra-individual titer changes over time (Fig. 2b–d). While the neutralization of B.1 (Fig. 2b) and of the Delta VoC (Fig. 2d) decreased significantly over time, the time dependent reduction was less pronounced for Beta (Fig. 2c) or Omicron BA.1 (Fig. 2e). Moreover, all Omicron BA.1 responders identified early after vaccination had still detectable neutralizing capacity at the late time points and two additional responders were identified in the late phase only (Fig. 2e). Therefore, the differences in neutralization titers between B.1 and Omicron BA.1 responders were less pronounced at the late time points than at peak response (Table 2).

In mid-2022, the initial BA.1 variant of the Omicron sub-lineage had further developed into additional variants such as BA.2, BA.2.12.1, BA.3, BA.4 and BA.5. To complete our analysis, we measured a sera sub-set with SARS-CoV-2 pseudotypes expressing the respective Spike variants resulting in comparable response patterns at peak response as seen for the Spike BA.1 SARS-CoV-2 pseudotype (Supplementary Fig. S1, Supplementary Table S5-S8). In sum, when looking at performance of primary vaccination protocols, immunization with homologous mRNA-1273 resulted in the highest responder rate across all Omicron sub-lineages, Ad26.CoV2.S in the lowest within the vaccination peak response phase, and a longitudinal follow-up showed that Omicron responses, while reduced, can be rather durable if present in the first place. Notably, we did not observe any significant differences in neutralization titers across the tested Omicron sub-variants. However, when looking at responder rates there is a tendency for slightly increased immune escape for BA.3 and BA.4/5.

## Discussion

We provide a comprehensive overview of neutralization responses from all currently available COVID-19 vaccination schemes in the European Union and the UK not only towards the Omicron BA.1 VoC, but also towards Beta and Delta VoC compared to the parental strain B.1. We expand on previous findings^12,17,30^ that neutralization towards Omicron including its sub-variants is particular poor after vaccination with vector-based formulations even within the peak phase shortly after vaccination and also provide data against clinically relevant Omicron sub-variants in a subset of our primary vaccinated cohort. Also consistent with other reports^16,23^, we observed a still surprisingly low cross-neutralization in BNT162b2 recipients. While some time differences exist between doses for homologous and heterologous vaccines, sampling periods after the complete scheme were within peak response making an earlier waning of BNT162b2-induced antibodies highly unlikely. While age impacts antibody titers and neutralization potency^31–33^, our group of BNT162b2 recipients is slightly younger compared to the other vaccinees making age an unlikely contributor to our observation. Additionally, no tendency became apparent for sex and comorbidities either. Considering our relatively small sample size, it is however possible that this low overall response in the BNT162b2 group was a spurious observation. Nevertheless, samples showing any cross-neutralizing responses early on remained responsive to Omicron BA.1 six months later. Notably, all mRNA-1273 recipients and 80% of those receiving any heterologous vaccination showed a detectable neutralization against Omicron BA.1 in our main analysis. It is not clear, why these vaccination protocols were more efficient against the Omicron BA.1 pseudotype than BNT162b2, but it is indicative that the baseline neutralization against the B.1 pseudotype was stronger in all of them in our sample cohorts.

The detectable responsiveness to the Omicron BA.1 pseudotype in all mRNA-1273 recipients differed from previous reports where usually several samples showed no measurable neutralization against Omicron BA.1^12,16,34^. This might be due to sampling differences, a result of increased sensitivity in our assay (as shown by consistent detection of the international standard within our study), or both. We chose responder rates as primary outcome because this is a less biased expression than fold changes if titers from non-responsive individuals are calculated. For the same reason, we used a non-parametric assay to evaluate differences, allowing us to include samples that were below detection threshold, but obviously very low in titer. Fold changes were calculated separately on a subset of samples that showed detectable titers in all circumstances. We observed an approximately 10–15-fold reduction in most vaccination regimens except BNT162b2, adding evidence that Omicron BA.1 cross-neutralization was impaired in this cohort. Our studies has several limitations. First, sample numbers in our cohort are relatively low. They are however comparable to the majority of other studies, are well-matched on age and sex and provide additional medical information such as pre-existing conditions and detailed information on vaccination time points not present in the earlier studies. Second, while our mean 55 day sampling time period after Ad26.CoV2.S administration is slightly longer than the four week sampling interval of the other vaccination schemes, our results are in line with others who reported poor Omicron BA.1 neutralization following vaccination with Ad26.CoV2.S both at one and five months after dosing^12,17,30^. Third, while self-reported information about a previous SARS-CoV-2 infection or vaccination can impact study outcome, two recent publications found a 98% consistency for vaccination type and 95% for vaccination date between self-reported and administrative records or a positive predictive value of 98.2% and a negative predictive value of 97.3% between a self-reported vaccination and the detection of SARS CoV-2 antibodies^35,36^. Fourth, we only examine vaccination-induced antibody titers, impact of cellular immunity on protection from Omicron infection is still poorly understood. Additionally, we omitted from our study people with previous infections or boosters. Others have however by now expanded on this and shown that mRNA vaccine boosters positively impact on levels of Omicron neutralizing antibodies^37–39^. Additionally, different vaccine types such as the inactivated whole virus formulation CoronaVac were also studied by others revealing that those do not show superior performance compared to mRNA-based vaccines^40–45^. Lastly, we utilized pseudo-virus-based assays for our study which only assess Spike-antibody mediated neutralization and not those of other surface-exposed structural proteins, if a live SARS-CoV-2 neutralization assay would be used. Nevertheless, the Spike protein is the most immuno-dominant antigen of SARS-CoV-2 and the sole antigen in most currently approved vaccines (except inactivated vaccines). Furthermore, no significant differences have been found between assays utilizing pseudo-virus or live-virus based assays in several studies^46,47^.

Following current recommendations, a booster vaccination is generally advised after six months in many industrialized countries, however in large parts of the world, even primary vaccination schemes continue to not even be available for the majority of the population^48^, making our data valuable to select the best performing immunization protocol for those settings. Although our results do not necessarily predict failure of vaccine effectiveness and omit measuring cellular immunity or non-neutralizing antibody effects, it does however suggest that active protection against infection with the Omicron VoCs may be reduced in many vaccinated individuals. Hence, booster vaccination are advisable at earlier stages, especially for at-risk groups in the absence of a precise and clinically relevant correlate of protection.

Overall, we provide further evidence that that amino acid mutations accumulated in the BA.1 Spike protein serve to escape vaccine-induced protection against infection after primary immunization schemes. Although more conclusive data on Omicron sub-variant infectivity and disease severity is available in mid-2022 than compared to December 2021, when our data was first generated, our data reconfirms the need to develop adapted second generation vaccines, distribute booster doses to at-risk groups to maintain levels of vaccine-induced protection against severe COVID-19 disease, and warrants careful monitoring of future variants of concern with comparable escape mutations, but increased disease severity.

## Supplementary Information

Supplementary Information 1.

Supplementary Information 2.

## Data Availability

All data generated or analyzed during this study are included in this published article and its supplementary information files.
