# Supplementary material for: Diminished neutralization responses towards SARS-CoV-2 Omicron VoC after mRNA or vector-based COVID-19 vaccinations
Source: Sci Rep. 2022 Nov 18;12:19858. doi: 10.1038/s41598-022-22552-y (PMC9673895; doi:10.1038/s41598-022-22552-y)
Supplement: Supplementary file 1 — Supplementary Information 1. [file 41598_2022_22552_MOESM1_ESM.docx]

**Supplementary Material - Diminished neutralization responses towards SARS-CoV-2 Omicron VoC after mRNA or vector-based COVID-19 vaccinations**

Henning Jacobsen^#,1^, Monika Strengert^#,2,3^, Henrike Maaß*^,1^, Mario Alberto Ynga Durand*^,1^, Maeva Katzmarzyk*^,1^, Barbora Kessel^2^, Manuela Harries^2^, Ulfert Rand^1^, Leila Abassi^1^, Yeonsu Kim^1^, Tatjana Lüddecke^1^, Kristin Metzdorf^1^, Pilar Hernandez^2^, Julia Ortmann^2^, Jana-Kristin Heise^2^, Stefanie Castell^2^, Daniela Gornyk^2^, Stephan Glöckner^2^, Vanessa Melhorn^2^, Yvonne Kemmling^2^, Berit Lange^2,5^, Alex Dulovic^4^, Patrick Marsall^4^, Julia Häring^4^, Daniel Junker^4^, Nicole Schneiderhan-Marra^4^, Markus Hoffmann^6,7^, Stefan Pöhlmann^6,7^, Gérard Krause^2,3,5,§^, Luka Cicin-Sain^1,5,8,§^

1 Helmholtz Centre for Infection Research, Department of Viral Immunology, Braunschweig, Germany

2 Helmholtz Centre for Infection Research, Department of Epidemiology, Braunschweig, Germany

3 TWINCORE, Centre for Experimental and Clinical Infection Research, a joint venture of the Hannover Medical School and the Helmholtz Centre for Infection Research, Hannover, Germany

4 NMI Natural and Medical Sciences Institute at the University of Tübingen, Reutlingen, Germany

5 German Centre for Infection Research (DZIF), partner site Hannover-Braunschweig, Braunschweig, Germany

6 Deutsches Primatenzentrum, Leibniz-Institut für Primatenforschung, Göttingen, Germany

7 Faculty of Biology and Psychology, Georg-August-University Göttingen, Germany

8 Centre for Individualized Infection Medicine (CIIM), a joint venture of Helmholtz Centre for Infection Research and Medical School Hannover, Germany

# (first authors), * (second authors), ^§^ (corresponding authors): these authors contributed equally.

Corresponding authors contact details:

Luka Cicin-Sain, Phone number: +49 (0)53161814616, Email address: [luka.cicin-sain@helmholtz-hzi.de](mailto:luka.cicin-sain@helmholtz-hzi.de), Postal address: Inhoffenstraße 7, 38124 Braunschweig, Germany.

**Supplementary Table S1. Comorbidities of all study participants**

(NA: not available; CVD: cardiovascular disease)

| **Sample cohort (n)** | **Comorbidities**  **(n, %)** | **CVD**  **(n, %)** | **Hyper-tension**  **(n, %)** | **Lung disease**  **(n, %)** | **Cancer**  **(n, %)** | **Diabetes (n, %)** | **Immuno-suppression**  **(n, %)** |
| --- | --- | --- | --- | --- | --- | --- | --- |
| one-dose Ad26.CoV2.S (23) | 0: 14 (60.9)  1: 6 (26.1)  2: 1 (4.3)  >2: 2 (8.7) | 3 (13.0) | 7 (30.4) | 2 (8.7) | 0 (0.0) | 1 (4.3) | 3 (13.0) |
| two-dose AZD1222 (25) | 0: 11 (44.0)  1: 10 (40.0)  2: 3 (12.0)  >2: 1 (4.0) | 3 (12.0) | 10 (40.0) | 2 (8.0) | 1 (4.0) | 3 (12.0) | 0 (0.0) |
| first dose AZD1222, second dose BNT162b2 (25) | 0: 13 (52.0)  1: 6 (24.0)  2: 5 (20.0)  >2: 1 (4.0) | 3 (12.0) | 12 (48.0) | 1 (4.0) | 1 (4.0) | 2 (8.0) | 0 (0.0) |
| first dose AZD1222, second dose mRNA-1273 (24) | 0: 11 (45.8)  1: 8 (33.3)  2: 5 (20.8)  >2: 0 (0.0) | 4 (16.7) | 11 (45.8) | 0 (0.0) | 1 (4.2) | 2 (8.3) | 0 (0.0) |
| two-dose mRNA-1273 (24) | 0: 15 (62.5)  1: 7 (29.2)  2: (8.3)  >2: 0 (0.0) | 1 (4.2) | 5 (20.8) | 3 (12.5) | 1 (4.2) | 1 (4.2) | 0 (0.0) |
| two-dose BNT162b2 T1 * (23) | 0: 13 (61.9)  1:6 (28.6)  2:2 (9.5)  >2: 0 (0.0)  2 NA | 1 (4.8) | 8 (38.1) | 1 (7.1) | 0 (0.0) | 0 (0.0) | 0 (0.0) |
| two-dose BNT162b2 T2 * (23) | 0: 14 (60.9)  1: 6 (26.1)  2: 3 (13.0)  >2: 0 (0.0) | 2 (8.7) | 7 (30.4) | 2 (8.7) | 0 (0.0) | 0 (0.0) | 2 (8.7) |
| * Two-dose BNT162b2 T1 and two-dose BNT162b2 T2 are paired longitudinal samples. | | | | | | | |

**Supplementary Table S2. Amino acid mutations of Spike proteins used for SARS-CoV-2 pseudotype construction compared to the parental strain B.1**

Shared mutations among the constructs are highlighted in bold.

| **B.1.351 (Beta)**  **(EPI_ISL_700428)** | **B.1.617.2 (Delta)**  **(EPI_ISL_1921353)** | **BA.1 (Omicron)**  **(EPI_ISL_6640919)** | **BA.2 (Omicron)**  **(EPI_ISL_8738174)** | **BA.2.12.1 (Omicron)**  **(EPI_ISL_12028907)** | **BA.3 (Omicron)**  **(EPI_ISL_8801154)** | **BA.4/5 (Omicron)**  **(EPI_ISL_11550739, EPI_ISL_12029894)** |
| --- | --- | --- | --- | --- | --- | --- |
|  | **T19R** |  | **T19I** | **T19I** |  | **T19I** |
|  |  |  | **L24S** | **L24S** |  | **L24S** |
|  |  |  | **25-27_del** | **25-27_del** |  | **25-27_del** |
|  |  | **A67V** |  |  | **A67V** |  |
|  |  | **69-70del** |  |  | **69-70del** | **69-70del** |
| D80A |  |  |  |  |  |  |
|  |  | **T95I** |  |  | **T95I** |  |
|  | **G142D** | **G142D** | **G142D** | **G142D** | **G142D** | **G142D** |
|  |  | **143-145del** |  |  | **143-145del** |  |
|  | E156G |  |  |  |  |  |
|  | 157-158del |  |  |  |  |  |
|  |  | **N211del/L212I** |  |  | **N211del/L212I** |  |
|  |  |  | **V213G** | **V213G** |  | **V213G** |
|  |  | Ins214EPE |  |  |  |  |
| 242-244del |  |  |  |  |  |  |
| R246I |  |  |  |  |  |  |
|  |  | **G339D** | **G339D** | **G339D** | **G339D** | **G339D** |
|  |  | **S371L** | **S371F** | **S371F** |  | **S371F** |
|  |  | **S373P** | **S373P** | **S373P** |  | **S373P** |
|  |  | **S375F** | **S375F** | **S375F** |  | **S375F** |
|  |  |  | **T376A** | **T376A** |  | **T376A** |
|  |  |  | **D405N** | **D405N** |  | **D405N** |
|  |  |  | **R408S** | **R408S** |  | **R408S** |
| **K417N** |  | **K417N** | **K417N** | **R408S** |  | **R408S** |
|  |  | **N440K** | **N440K** | **N440K** | **N440K** | **N440K** |
|  |  | G446S |  |  |  |  |
|  | **L452R** |  |  | **L452Q** |  | **L452R** |
|  |  | **S477N** | **S477N** | **S477N** | **S477N** | **S477N** |
|  | **T478K** | **T478K** | **T478K** | **T478K** | **T478K** | **T478K** |
| **E484K** |  | **E484A** | **E484A** | **E484A** | **E484A** | **E484A** |
|  |  |  |  |  |  | E486V |
|  |  | **Q493R** | **Q493R** | **Q493R** | **Q493R** |  |
|  |  | G496S |  |  |  |  |
|  |  | **Q498R** | **Q498R** | **Q498R** | **Q498R** | **Q498R** |
| **N501Y** |  | **N501Y** | **N501Y** | **N501Y** | **N501Y** | **N501Y** |
|  |  | **Y505H** | **Y505H** | **Y505H** | **Y505H** | **Y505H** |
|  |  | T547K |  |  |  |  |
| **D614G** | **D614G** | **D614G** | **D614G** | **D614G** | **D614G** | **D614G** |
|  |  | **H655Y** | **H655Y** | **H655Y** | **H655Y** | **H655Y** |
|  |  | **N679K** | **N679K** | **N679K** | **N679K** | **N679K** |
|  | **P681R** | **P681H** | **P681H** | **P681H** | **P681H** | **P681H** |
| A701V |  |  |  |  |  |  |
|  |  |  |  | S704L |  |  |
|  |  | **N764K** | **N764K** | **N764K** | **N764K** | **N764K** |
|  |  | **D796Y** | **D796Y** | **D796Y** | **D796Y** | **D796Y** |
|  |  | N856K |  |  |  |  |
|  | D950N |  |  |  |  |  |
|  |  | **Q954H** | **Q954H** | **Q954H** | **Q954H** | **Q954H** |
|  |  | **N969K** | **N969K** | **N969K** | **N969K** | **N969K** |
|  |  | L981F |  |  |  |  |

**Supplementary Table S3. Spike B.1- and RBD B.1-antibody titres and responder rates determined by MULTICOV-AB* of all participants**

| **Sample cohort (n)** | **Responder rate**  **MULTICOV-AB****  **(n, %)** | **GMT Spike B.1 S/CO (95% CI)** | **Spearman’s ρ: FRNT_50_ B.1 – Spike B.1 S/CO** | **GMT RBD B.1**  **S/CO (95% CI)** | **Spearman’s ρ: FRNT_50_ B.1 – RBD B.1 S/CO** |
| --- | --- | --- | --- | --- | --- |
| one-dose Ad26.CoV2.S (23) | 19 (82.6) | 2.78  (1.84-4.18) | 0.619 | 3.63  (2.30-5.73) | 0.485 |
| two-dose AZD1222 (25) | 25 (100.0) | 5.34  (4.35-6.57) | 0.765 | 9.46  (7.21-12.40) | 0.852 |
| first dose AZD1222, second dose BNT162b2 (25) | 25 (100.0) | 12.27  (10.90-13.81) | 0.640 | 22.10  (18.39-26.55) | 0.639 |
| first dose AZD1222, second dose mRNA-1273 (24) | 24 (100.0) | 12.03  (10.29-14.07) | 0.520 | 23.43  (18.96-28.96) | 0.870 |
| two-dose mRNA-1273 (24) | 24 (100.0) | 13.52  (13.15-13.91) | 0.351 | 29.87  (28.71-31.09) | 0.461 |
| two-dose BNT162b2 T1 *** (23) | 23 (100.0) | 11.83  (9.82-14.24) | 0.780 | 23.93  (18.64-30.72) | 0.880 |
| two-dose BNT162b2 T2 *** (23) | 22 (95.7) | 6.67  (5.26-8.45) | 0.840 | 7.28  (5.12-10.36) | 0.893 |
| * Previously published as part of Front. Immunol., 16/02/2022: https://doi.org/10.3389/fimmu.2022.828053  “Comparative Magnitude and Persistence of Humoral SARS-CoV-2 Vaccination Responses in the Adult Population in Germany”.  ** Responders are classified based on a dual IgG S/CO of ≥ 1.0 for both Spike parental B.1 and RBD B.1 antigens in MULTICOV-AB.  *** Two-dose BNT162b2 T1 and two-dose BNT162b2 T2 are paired longitudinal samples. | | | | | |

**Supplementary Table S4. Geometric means of responses and fold reduction in all samples**

| **Sample cohort (n)** | **GMT (95% CI) of all samples across indicated variants** | | | |
| --- | --- | --- | --- | --- |
|  | **B.1** | **Beta B.1.351** | **Delta B.1.617.2** | **Omicron BA.1** |
| one-dose Ad26.CoV2.S (23) | 8.67  (4.05-18.56) | 1.48  (0.83-2.63) | 4.72  (2.31-9.63) | 1.28  (0.82-1.98) |
| two-dose AZD1222 (25) | 89.93  (58.84-137.40) | 14.92  (7.63-29.20) | 38.21  (21.0-69.33) | 4.45  (2.41-8.21) |
| first dose AZD1222, second dose BNT162b2 (25) | 611.80  (412.60-907.30) | 83.39  (43.47-160.00) | 336.30  (211.40-535.00) | 26.61  (13.48-52.56) |
| first dose AZD1222, second dose mRNA-1273 (24) | 724.60  (411.7-1275.00) | 140.20  (57.53-341.90) | 474.60  (249.60-902.50) | 45.59  (22.64-91.82) |
| two-dose mRNA-1273 (24) | 453.80  (351.20-586.40) | 85.48  (55.45-131.80) | 393.50  (284.40-544.40) | 29.50  (21.58-40.33) |
| two-dose BNT162b2 T1 ** (23) | 159.00  (86.37-292.70) | 21.27  (11.47-39.44) | 151.80  (84.80-271.90) | 4.01  (2.06-7.81) |
| two-dose BNT162b2 T2 ** (23) | 45.41  (26.57-77.62) | 12.59  (6.46-24.53) | 41.70  (24.72-70.35) | 4.48  (2.42-8.30) |
| **Sample cohort (n)** | **Fold reduction in GMT of all samples across indicated variants** | | | |
|  | **B.1** | **Beta B.1.351** | **Delta B.1.617.2** | **Omicron BA.1** |
| one-dose Ad26.CoV2.S (23) | - | 5.90 | 1.84 | 6.79 |
| two-dose AZD1222 (25) | - | 6.03 | 2.35 | 20.20 |
| first dose AZD1222, second dose BNT162b2 (25) | - | 7.34 | 1.82 | 22.99 |
| first dose AZD1222, second dose mRNA-1273 (24) | - | 5.17 | 1.53 | 15.89 |
| two-dose mRNA-1273 (24) | - | 5.31 | 1.15 | 15.38 |
| two-dose BNT162b2 T1 ** (23) | - | 7.48 | 1.05 | 39.69 |
| two-dose BNT162b2 T2 ** (23) | - | 3.61 | 1.09 | 10.13 |
| ** Two-dose BNT162b2 T1 and two-dose BNT162b2 T2 are paired longitudinal samples. | | | | |

**Supplementary Table S5. Sample characteristics of sera subset additionally measured with** **Omicron sub-lineages BA.2, BA2.12.1, BA.3 and BA.4/5 pseudotypes**

(na: not applicable; CVD: cardiovascular disease)

| **Sample cohort (n)** | **ΔT (days) post-complete vaccination (mean, SD)** | **ΔT (days) between doses**  **(mean, SD)** | **Age (years), median (IQR)** | **Sex (n, %)** | | **Comorbidities**  **(n, %)** | **CVD**  **(n, %)** | **Hyper-tension**  **(n, %)** | **Lung disease**  **(n, %)** | **Cancer**  **(n, %)** | **Diabetes (n, %)** | **Immuno-suppression**  **(n, %)** |
| --- | --- | --- | --- | --- | --- | --- | --- | --- | --- | --- | --- | --- |
|  |  |  |  | **Female** | **Male** |  |  |  |  |  |  |  |
| one-dose Ad26.CoV2.S (13) | 52.5 (22.4) | na | 57  (38-72) | 8 (61.5) | 5 (38.5) | 0: 7 (53.8)  1: 4 (30.8)  2: 1 (7.7)  >2: 1 (7.7) | 2 (15.4) | 4 (30.8) | 1 (7.7) | 0 (0.0) | 0 (0.0) | 2 (15.4) |
| two-dose AZD1222 (11) | 30.7 (2.7) | 75.6 (4.1) | 64  (61-65) | 7 (63.6) | 4 (36.4) | 0: 4 (36.4)  1: 4 (36.4)  2: 2 (18.2)  >2: 1 (9.1) | 3 (27.3) | 4 (36.4) | 2 (18.2) | 1 (9.1) | 1 (9.1) | 0 (0.0) |
| first dose AZD1222, second dose BNT162b2 (10) | 24.2 (8.3) | 62.7 (23.0) | 60  (55-70) | 7 (70.0) | 3 (30.0) | 0: 5 (50.0)  1: 3 (30.0)  2: 2 (20.0)  >2: 0 (0.0) | 2 (20.0) | 5 (50.0) | 0 (0.0) | 0 (0.0) | 0 (0.0) | 0 (0.0) |
| first dose AZD1222, second dose mRNA-1273 (13) | 18.9 (15.9) | 72.2 (9.5) | 67  (65-71) | 8 (61.5) | 5 (38.5) | 0: 4 (30.8)  1: 5 (38.5)  2: 4 (30.8)  >2: 0 (0.0) | 2 (15.4) | 8 (61.5) | 0 (0.0) | 1 (7.7) | 2 (15.4) | 0 (0.0) |
| two-dose mRNA-1273 (9) | 37.0 (2.2) | 28.0 (0.0) | 61  (55-70) | 5 (55.6) | 4 (44.4) | 0: 4 (44.4)  1: 3 (33.3)  2: 2 (22.2)  >2: 0 (0.0) | 0 (0.0) | 4 (44.4) | 2 (22.2) | 0 (0.0) | 1 (11.1) | 0 (0.0) |
| two-dose BNT162b2 T1 * (9) | 30.9 (10.0) | 21.0 (0.0) | 53  (47-59) | 7 (77.8) | 2 (22.2) | 0: 6 (66.7)  1: 2 (22.2)  2: 1 (11.1)  >2: 0 (0.0) | 1 (11.1) | 3 (33.3) | 0 (0.0) | 0 (0.0) | 0 (0.0) | 0 (0.0) |
| two-dose BNT162b2 T2 * (9) | 173.0 (18.1) |  | 53  (47-60) |  |  | 0: 7 (77.8)  1: 1 (11.1)  2: 1 (11.1)  >2: 0 (0.0) | 1 (11.1) | 2 (22.2) | 0 (0.0) | 0 (0.0) | 0 (0.0) | 0 (0.0) |
| * Two-dose BNT162b2 T1 and two-dose BNT162b2 T2 are paired longitudinal samples. | | | | | | | | | | | | |

**Supplementary Table S6. Serological characterization of participant’s sera subset measured with additional Omicron sub-lineages BA.2, BA2.12.1, BA.3 and BA.4/5 pseudotypes**

| **Sample cohort (n)** | **Responder rate**  **MULTICOV-AB***  **(n, %)** | **GMT Spike B.1 S/CO (95% CI)** | **Spearman’s ρ: FRNT_50_ B.1 – Spike B.1 S/CO** | **GMT RBD B.1**  **S/CO (95% CI)** | **Spear-man’s ρ: FRNT_50_ B.1 – RBD B.1 S/CO** | **Responder rates FRNT_50_ assay for indicated variants (n; %)** | | | | | | | | **Paired responder samples across all variants in cohort (n; [% of total n per cohort])** |
| --- | --- | --- | --- | --- | --- | --- | --- | --- | --- | --- | --- | --- | --- | --- |
|  |  |  |  |  |  | **B.1** | **B. 1.351** | **B.1.617.2** | **BA.1** | **BA.2** | **BA.2.12.1** | **BA.3** | **BA.4/5** |  |
| one-dose Ad26.CoV2.S (13) | 10 (76.9) | 2.78  (1.43-5.41) | 0.730 | 3.62  (1.72-7.64) | 0.635 | 9 (69.2) | 1 (7.7) | 8 (61.5) | 2 (15.4) | 1 (7.7) | 1 (7.7) | 1 (7.7) | 1 (7.7) | 1 (7.7) |
| two-dose AZD1222 (11) | 11 (100.0) | 5.25  (3.67-7.52) | 0.673 | 8.98  (5.56-14.50) | 0.855 | 11 (100.0) | 9 (81.8) | 9 (81.8) | 6 (54.5) | 5 (45.5) | 5 (45.5) | 4 (36.4) | 4 (36.4) | 4 (36.4) |
| first dose AZD1222, second dose BNT162b2 (10) | 10 (100.0) | 12.52  (11.25-13.94) | 0.879 | 22.27  (17.55-28.25) | 0.806 | 10 (100.0) | 9 (90.0) | 10 (100.0) | 9 (90.0) | 9 (90.0) | 9 (90.0) | 9 (90.0) | 9 (90.0) | 9 (90.0) |
| first dose AZD1222,  second dose mRNA-1273 (13) | 13 (100.0) | 12.58  (11.32-13.98) | 0.407 | 24.50  (19.89-30.16) | 0.874 | 13 (100.0) | 12 (92.3) | 13 (100.0) | 12 (92.3) | 12 (92.3) | 12 (92.3) | 12 (92.3) | 12 (92.3) | 12 (92.3) |
| two-dose mRNA-1273 (9) | 9 (100.0) | 13.25  (12.65-13.89) | 0.167 | 1.09  (27.31-31.38) | 0.200 | 9 (100.0) | 9 (100.0) | 9 (100.0) | 9 (100.0) | 9 (100.0) | 9 (100.0) | 9 (100.0) | 7 (77.8) | 7 (77.8) |
| two-dose BNT162b2 T1 ** (9) | 9 (100.0) | 13.18  (12.12-14.32) | 0.683 | 27.92  (22.90-34.03) | 0.817 | 9 (100.0) | 9 (100.0) | 9 (100.0) | 7 (77.8) | 8 (88.9) | 8 (88.9) | 5 (55.6) | 5 (55.6) | 5 (55.6) |
| two-dose BNT162b2 T2 ** (9) | 9 (100.0) | 7.87  (6.30-9.82) | 0.867 | 9.99  (6.79-14.70) | 0.900 | 9 (100.0) | 8 (88.9) | 9 (100.0) | 7 (77.8) | 8 (88.9) | 7 (77.8) | 6 (66.7) | 5 (55.6) | 5 (55.6) |
| * Responders are classified based on a dual IgG S/CO of ≥ 1.0 for both Spike parental B.1 and RBD B.1 antigens in MULTICOV-AB.  ** Two-dose BNT162b2 T1 and two-dose BNT162b2 T2 are paired longitudinal samples. | | | | | | | | | | | | | | |

**Supplementary Table S7. Geometric means of responses and fold reduction in sera sub-sets measured additionally with Omicron sub-lineages BA.2, BA.2.12.1, BA.3 and BA.4/5 pseudotypes**

| **Sample cohort (n)** | **GMT (95% CI) of indicated variants for all samples** | | | | | | | | | | | | | | | **Paired responders across all variants in cohort (n; [% of total n])** | **GMT (95% CI) of paired responders across indicated variants** | | | | | | | |
| --- | --- | --- | --- | --- | --- | --- | --- | --- | --- | --- | --- | --- | --- | --- | --- | --- | --- | --- | --- | --- | --- | --- | --- | --- |
|  | **B.1** | **B. 1.351** | | **B.1.617.2** | | **BA.1** | | **BA.2** | | **BA.2.12.1** | | **BA.3** | | **BA.4/5** | |  | **B.1** | **B.1.351** | **B.1.617.2** | **BA.1** | **BA.2** | **BA.2.12.1** | **BA.3** | **BA.4/5** |
| one-dose Ad26.CoV2.S (13) | 9.60  (2.84-32.42) | 1.54  (0.60-  3.91) | | 5.37  (1.64-  17.58) | | 1.540  (0.67  -3.46) | | 1.45  (0.65  -3.26) | | 1.56  (0.60-4.10) | | 1.426  (0.66-  3.10) | | 1.46  (0.64-3.30) | | 1 (7.7)* | na | na | na | na | na | na | na | na |
| two-dose AZD1222 (11) | 62.98  (32.03-  123.80) | 13.76  (4.84-  39.10) | | 24.64  (7.81-  77.78) | | 4.24  (1.54-  11.66) | | 4.23  (1.32-  13.54) | | 4.30  (1.34-  13.81) | | 2.72  (1.02-  7.25) | | 2.48  (1.04-  5.88) | | 2 (18.2)** | na | na | na | na | na | na | na | na |
| first dose AZD1222,second dose BNT162b2 (10) | 544.10  (264.10-1121.00) | 58.14  (17.17-  196.90) | | 301.90  (154.10-  591.10) | | 26.07  (8.64-  78.66) | | 29.52  (10.46-  83.29) | | 30.96  (10.99-  87.17) | | 18.69  (7.18-  48.66) | | 21.46  (7.68-  59.95) | | 9 (90.0) | 664.90  (350.20-  1263.0) | 91.32  (42.70  195.30) | 355.00  (186.80-  674.50) | 37.46  (16.12-87.06) | 43.00  (21.87-  84.55) | 45.33  (23.62-  87.02) | 25.87  (12.88-  51.97) | 30.17  (13.91-  65.44) |
| first dose AZD1222,  second dose mRNA-1273 (13) | 773.10  (423.20-  1413.00) | 153.60  (54.64-  431.90) | | 497.90  (238.20-  1041.00) | | 57.79  (23.74-  140.7) | | 56.98  (24.15-  134.50) | | 62.67  (26.29-  149.40) | | 36.85  (16.88-  80.46) | | 37.72  (16.34-  87.10) | | 12 (92.3) | 954.20  (620.90-1466.0) | 233.70  (137.50-  397.00) | 657.6  (414.3-  1044.0) | 81.04  (46.86-  140.20) | 79.81  (48.95-  130.10) | 88.48  (54.81-  142.80) | 49.77  (31.21-  79.39) | 51.04  (28.98-  89.92) |
| two-dose mRNA-1273 (9) | 320.20  (247.20-  414.70) | 43.58  (25.12-  75.60) | | 261.20  (199.40-342.10) | | 21.21  (14.77-  30.44) | | 23.26  (15.47-  34.98) | | 23.25  (14.68-  36.82) | | 15.46  (10.43-  22.92) | | 8.46  (3.10-  23.18) | | 7 (77.8) | 322.80  (234.40-  444.6) | 44.04  (20.70-  93.72) | 267.50  (198.50-360.60) | 24.59  (16.61-  36.39) | 26.99  (16.94-  43.00) | 27.75  (16.58-  46.44) | 18.81  (13.36-  26.48) | 15.58  (9.12-  26.61) |
| two-dose BNT162b2 T1 (9) | 256.50  (128.50-  512.20) | 37.86  (23.34-  61.42) | | 205.60  (106.70  -396.30) | | 10.44  (3.37-  32.37) | | 14.58  (5.90-  36.03) | | 15.20  (6.04-  38.20) | | 5.22  (1.52-  17.95) | | 4.94  (1.49-  16.41) | | 4 (44.4) | 485.50  (289.30-  814.80) | 65.73  (32.21-  134.10) | 391.30  (156.10-981.00) | 35.77  (23.71-53.97) | 35.75  (22.93-  55.73) | 37.82  (22.14-  64.59) | 23.56  (14.59-38.06) | 19.69  (8.17-47.46) |
| two-dose BNT162b2 T2 (9) | 91.34  (46.55-  179.20) | 20.46  (6.91-  60.62) | | 64.99  (32.10-  131.70) | | 9.20  (3.14-  26.91) | | 10.36  (4.78-  22.44) | | 8.98  (3.22-  25.07) | | 5.30  (1.95-  14.37) | | 4.08  (1.44-  11.58) | | 4 (44.4) | 179.10  (74.36-  431.20) | 55.28  (15.43-  198.00) | 123.6  (29.54-  517.10) | 22.77  (6.20-  83.65) | 21.12  (12.13-  36.80) | 24.90  (14.33-  43.27) | 13.62  (5.83-  31.83) | 13.49  (7.75-  23.50) |
| **Sample cohort (n)** | **Fold reduction in GMT indicated variants for all samples** | | | | | | | | | | | | | | | **Paired responders across all variants in cohort (n; [% of total n])** | **Fold reduction in GMT of paired responders across indicated variants** | | | | | | | |
|  | **B.1** | | **B. 1.351** | | **B.1.617.2** | | **BA.1** | | **BA.2** | | **BA.2.12.1** | | **BA.3** | | **BA.4/5** |  | **B.1** | **B.1.351** | **B.1.617.2** | **BA.1** | **BA.2** | **BA.2.12.1** | **BA.3** | **BA.4/5** |
| one-dose Ad26.CoV2.S (13) | - | | 6.25 | | 1.79 | | 6.23 | | 6.62 | | 6.16 | | 6.73 | | 6.59 | 1 (7.7) * | - | na | na | na | na | na | na | na |
| two-dose AZD1222 (11) | - | | 4.58 | | 2.56 | | 14.84 | | 14.88 | | 14.66 | | 23.13 | | 25.44 | 2 (18.2) ** | - | na | na | na | na | na | na | na |
| first dose AZD122,. second dose BNT162b2 (10) | - | | 9.36 | | 1.80 | | 20.87 | | 18.43 | | 17.57 | | 29.11 | | 25.35 | 9 (90.0) | - | 7.28 | 1.87 | 17.75 | 15.46 | 14.67 | 25.70 | 22.04 |
| first dose AZD1222,second dose mRNA-1273 (13) | - | | 5.03 | | 1.55 | | 13.38 | | 13.57 | | 12.34 | | 20.98 | | 20.50 | 12 (92.3) | - | 4.08 | 1.45 | 11.77 | 11.96 | 10.78 | 19.17 | 18.70 |
| two-dose mRNA-1273 (9) | - | | 7.35 | | 1.23 | | 15.10 | | 13.77 | | 13.77 | | 20.71 | | 37.83 | 7 (77.8) | - | 7.33 | 1.21 | 13.13 | 11.96 | 11.63 | 17.16 | 20.72 |
| two-dose BNT162b2 T1 (9) | - | | 6.77 | | 1.25 | | 24.57 | | 17.59 | | 16.88 | | 49.10 | | 51.90 | 4 (44.4) | - | 7.39 | 1.24 | 13.57 | 13.58 | 12.84 | 20.61 | 24.66 |
| two-dose BNT162b2 T2 (9) | - | | 4.46 | | 1.41 | | 9.93 | | 8.82 | | 10.17 | | 17.25 | | 22.38 | 4 (44.4) | - | 3.24 | 1.45 | 7.87 | 8.48 | 7.19 | 13.15 | 13.28 |
| *,** No further analysis was performed based on the paired responder number of 1 or 2 individuals across all VoCs tested for one-dose Ad26.CoV2.S and two-dose AZD1222 immunizations. | | | | | | | | | | | | | | | | | | | | | | | | |

**
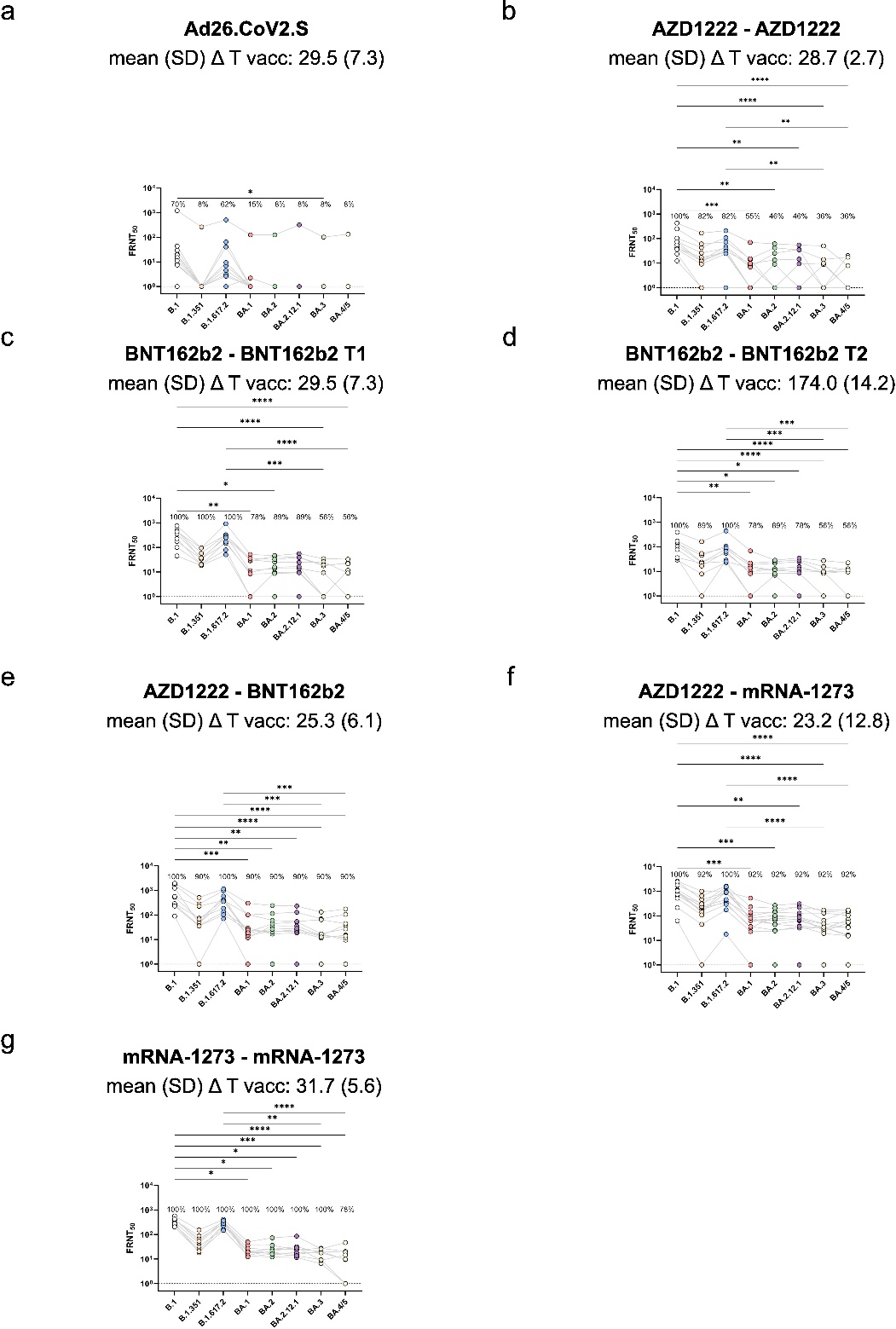
**

**Supplementary Fig 1. Impact of SARS-CoV-2 vaccination schemes on neutralization response towards Omicron sub-lineages BA.2, BA2.12.1, BA.3 and BA.4/5.**

Vaccination-induced neutralization potency against Omicron sub-lineages BA.2, BA2.12.1, BA.3 and BA.4/5 was measured in individuals who received a vector-based vaccination with single dose Ad26.CoV2.S (n=13, a), two doses of AZD1222 (n=11, b), two doses of mRNA vaccine BNT162b2 after T1 and T2 (n=9, c, d), a heterologous two-dose vaccination with AZD1222-BNT162b2 (n=10, e) or AZD1222-mRNA-1273 (n=13, f) or two doses of mRNA vaccine mRNA-1273 (n=9, g) after the indicated time periods following the last dose. FRNT_50_ data is expressed for each serum sample. Interconnecting lines represent sample data from the same donor. Non-neutralizing sample values were arbitrarily set to 1 for presentation purposes indicated by a dashed line. FRNT_50_ values for Omicron BA.1, B.1.351, B.1.617.2 and Wuhan B.1 pseudotypes are again displayed for clarity and comparison purposes. Percentage responder rates, FRNT_50_ geometric mean titres (GMT) and fold-change in neutralization potency per SARS-CoV-2 pseudotype are separately listed in Supplementary Table S7. Time between sampling and full vaccination in days is displayed as mean and SD below the vaccination scheme. BNT162b2 T1 and BNT162b2 T2 are paired longitudinal samples.
